# The Course and the Effects of Agricultural Biomass Pyrolysis in the Production of High-Calorific Biochar

**DOI:** 10.3390/ma15031038

**Published:** 2022-01-28

**Authors:** Paweł Kazimierski, Katarzyna Januszewicz, Wojciech Godlewski, Aleksander Fijuk, Tomasz Suchocki, Patryk Chaja, Beata Barczak, Dariusz Kardaś

**Affiliations:** 1 Institute of Fluid Flow Machinery, Polish Academy of Sciences, 80-231 Gdańsk, Poland; wgodlewski@imp.gda.pl (W.G.); afijuk@imp.gda.pl (A.F.); tsuchocki@imp.gda.pl (T.S.); patryk.chaja@imp.gda.pl (P.C.); dk@imp.gda.pl (D.K.); 2 Department of Energy Conversion and Storage, Chemical Faculty, Gdańsk University of Technology, Narutowicza 11/12, 80-233 Gdańsk, Poland; katarzyna.januszewicz@pg.edu.pl (K.J.); s171325@student.pg.edu.pl (B.B.)

**Keywords:** waste biomass, pyrolysis, biomass fuel, charcoal, biochar

## Abstract

The thermal pyrolysis of agriculture biomass has been studied in a fixed-bed reactor, where the pyrolysis was conducted at a steady temperature of 800 °C. This work analyses the pyrolysis products of six agricultural wastes: pistachio husks, walnut husks, sunflower hulls, buckwheat husks, corncobs and coconut shells. The conducted research compared examples of large waste biomass streams from different parts of the world as a potential source of renewable energy. Additionally, the kinetics of the reaction with the activation energy were analyzed and calculated for all raw materials in pyrolysis process. Biochars are characterised by higher combustion heat in comparison to the raw material samples. The average value of the heat of combustion increased due to pyrolysis process from 10 MJ/kg, with minimal value of 2.7 MJ/kg (corncob) and maximum of 13.0 MJ/kg for coconut, which is also characterised by the maximal absolute combustion heating value (32.3 MJ/kg). The increase in calorific values varied from 15% to 172% (with 54% reference for wood chips), which indicates that charring is an effective method for increasing the energy concentration. The obtained biochar were compared with wood chips, which are widely used solid fuel of organic origin. The studied biomass-derived fuels are characterised by lower ash contribution than wood. An analogous observation was made for the obtained biochars, whose ash contribution was lower than for the chips in terms of both unit-mass and unit-combustion-heat. The main advantage of this method is the production of solid fuel from biomass, which increases the calorific value and bulk density of biochar in comparison to raw material.

## 1. Introduction

The authors assume that waste from the food industry is a potentially good raw material for the production of high-quality coals for energy purposes. Due to recent technological progress and the growth of the world’s population, the global primary energy consumption has been increasing for years. In 2018, the energy consumption growth reached 2.9%. In comparison, over the previous 10 years, it was 1.5% per year on average. According to Eurostat [1], global energy demand for industry is based mainly on the combustion of fossil fuels, especially oil and gas (80% of its feedstock and energy). In 2018, carbon dioxide emissions increased by 2% globally compared to the preceding year, which was the fastest growth in seven years. The consumption of fossil fuels is associated with the emission of gases and dust, which are harmful to human health [2,3,4,5,6] as well as the environment [7,8]. Meanwhile, the share of these fuels in the global energy balance is still dominant. At the same time, Renewable Energy Sources (RES) are experiencing growth in the global share of energy production [9]. This increase in RES in the global share in energy production is forced by many of the regulations that have been introduced worldwide. This share is targeted to reach at least 50% in the EU by 2030 [10]. Analyses of different measures aiming at transition to “clean technologies” were carried out [11].

To emphasise the importance of legal regulations for research and the development of a given type of fuel—in this case, biomass of agricultural origin—we use the example of the obligation to use a mass share of the biomass of agricultural origin in the overall biomass used to generate electricity introduced in 2018 in Poland. This regulation requires a total share of 85% for multi-fuel combustion installations and dedicated multi-fuel combustion installations with an installed electrical capacity greater than 5 MW. Furthermore, it requires 10% for dedicated biomass combustion installations and hybrid systems with an installed electrical capacity greater than 20 MW [12,13,14,15,16,17].

Biomass has the largest share of energy production among all RES globally [3,18]. One of the most widely used sources of biomass are by-products arising from various types of human activities, such as organic waste, sawdust or husks of various crops. Consequently, biomass can also help to reduce waste [19,20,21,22]. The analysis carried out by the World Bioenergy Association [23] presented the theoretical energy potential of agricultural residues that could meet up to 20% of global energy demand in 2014. Plants absorb carbon dioxide during growth and emit it during combustion. Energy generated from biomass during pyrolysis process is neutral, the plants appear either carbon-neutral or carbon-negative [24]. The most commonly used sources of biomass are woodchips, sawdust, pellets, briquettes, bark, straw, and plants grown for biomass from plantations such as energy willow, giant miscanthus, Virginia mallow or Jerusalem artichoke [25]. Many biofuels have negligible sulphur, nitrogen and ash content compared to fossil fuels, which lowers the emission of harmful substances, such as SOx, NOx, and soot [24,25]. However, biomass also has some disadvantages, such as a lower carbon content and calorific value than fossil fuels [25,26,27]. Lignocellulosic biomass (which mainly consists of hemicellulose, cellulose, and lignin, with varying thermal stabilities [28] is characterised by the rate of growth of 30–240 Barrel of Oil Equivalent (BOE) per hectare per year [29]. According to research conducted by Khan et al. [18], biomass and waste supply up to 10% of global current energy demand. It has been estimated that the full potential of the annual available biomass amounts to 1.08 × 1011 toe (tons of oil equivalent), which would mean ten times the current global energy demand [30,31].

Biomass can be transformed using chemical, biochemical, electrochemical and thermochemical conversions. Thermochemical conversions can be divided into torrefaction, gasification, combustion, and pyrolysis [26,27]. In the pyrolysis process, previously dried and prepared biomass is subjected to a temperature above its decomposition temperature in the absence of oxygen, which causes intensified breaking of chemical bonds in molecules [28]. This leads to fuels in solid (biochar) and volatile (liquids and gases) fractions [29]. In inert conditions and under the influence of heating, biomass emits organic vapours, which include fragments of cellulose, hemicellulose and lignin polymers [28]. When they are condensed, they can be used to produce bio-oil. The heat obtained during the combustion of non-condensable gases can be used as an energy source for pyrolysis. Biochars are carbon-rich process residues [29]. The main uses of biocarbons, in addition to being used as fuel [30,31], are filtration carbons [32], sorbents, porous materials [33], carbon electrodes [34]. Due to global trends, nowadays thermal energy from pyrolysis can be used for, among others, for the production of electricity [35,36].

The choice of pyrolysis as a process of materials’ treatment was selected due to several reasons; Neves et al. [37] examined the quantity and properties of the products obtained via this process, depending on the parameters of the experiment. They showed that in a low-temperature range (i.e., less than 500 °C), the solid biomass decomposes mainly into volatile substances. The decrease in the amount of char obtained as the temperature increases is compensated by the production of liquid products. Peters et al. [38] studied the potential of various pyrolysis methods to determine the optimal method to limit greenhouse gas emissions and the negative impact on the environment. The influence of increasing pyrolysis peak temperature on products was investigated thanks to the different volatilisation temperature ranges of biomass components [39]. The biochar that we examined in this paper was produced from agro-biomass (i.e., biomass obtained as waste from the agricultural industry).

The biomass used in the experimental work was chosen based on the main production in Poland and others countries; the amount of waste generated during food production was also taken into account. The comparison of the pyrolysis process of various types of biomass allows for the assessment of the proposed technology as useful on an industrial scale. An analysis of the pyrolysis process in the micro-scale and laboratory steel was performed, which allows to observe the desired effect of this process on the obtained biochar. The novelty in this work is the analysis of the kinetics of the reaction with the use of activation energy calculations and the comparison of these processes in six different types of waste biomass.

## 2. Experimental Part

### 2.1. Materials

**Figure 1:**
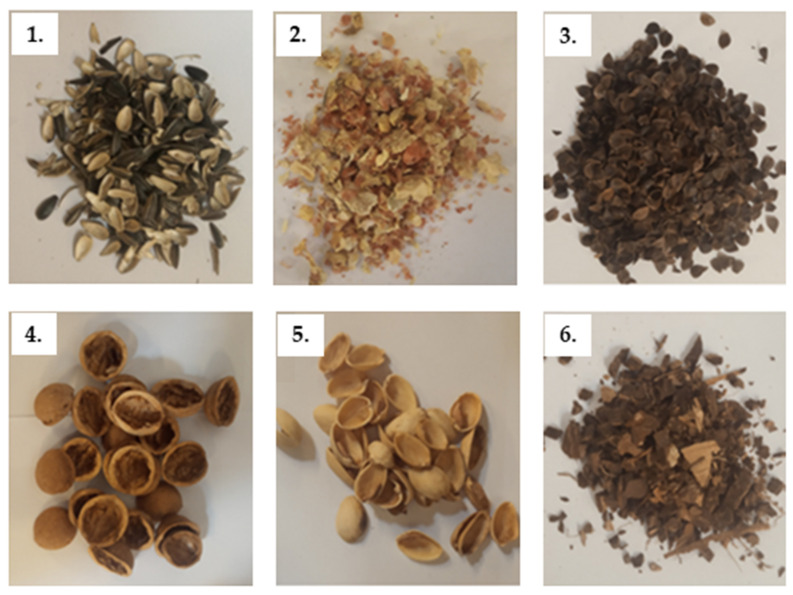
Raw materials: sunflower hulls (**1**), corncob (**2**), coconut shells (**3**), walnut husks (**4**), pistachio husks (**5**), buckwheat husks (**6**).

The experiment studied followed six biomass materials as potential eco-friendly substitutes for conventional fuels: pistachio husks (*Pistacia vera*), walnuts husks (*Juglandaceae*), sunflower husks (*Helianthus*), coconut shells (*Cocos nucifera*), corncobs (*Zea mays*), buckwheat husks (*Fagopyrum esculentum*). These materials are waste biomass (Figure 1) from industrial processes, which elevates their attractiveness as a prospective fuel due to abundance in areas of production, low price and easy access.

Pistachio husks come from pyrenes produced in the USA and coconut shells, which are the biomass waste from Philippines, whereas all of the other materials used in the experiments originate from Polish farms. Three of the substances (i.e., corncobs, coconut shells, walnut husks) were milled to a granularity of less than 5 mm to enable loading into the chamber in a packed fashion and to enhance the pyrolytic process. All of these materials are characterised by abundance, low acquisition price and easy access. These attributes, if related to a viable energy source, describe a potential candidate for substituting conventional fuels. Considering these properties, developing feasible and sustainable biomass-based power generation technology could be beneficial for both the manufacturers, due to elimination of disposal issues, and clients, because of their potential environment-friendliness and low price.

These waste materials are sourced in regions with a significant mass stream and energy source. Table 1 summarises the production of crops, estimated amount of waste from the crops, mass of waste, energy potential in waste and charcoal, and the amount of carbon and wood corresponding to the biomass stream of charcoal.

### 2.2. Methodology

Samples of every biomass species as raw material were pyrolyzed in a fixed-bed reactor. The process was carried out in a high-temperature muffle furnace, at a steady temperature of 800 °C. The sample of raw material (about 100 g) was put into a 250 mL closed hermetic reactor and were pyrolyzed. The large thermal inertia and simple design of the device enabled fast pyrolysis in steady conditions, as well as prompt abort of the pyrolysis by withdrawing and cooling of the sample. During the experiment, the furnace was heated to 800 °C and a thermostat was activated when the desired temperature was reached. Before and after the fuel sample was placed in the furnace, the chamber was insufflated with neutral gas. The samples were subjected to the conditions inside the chamber for 30 min. Additional pyrolysis process in microscale was conducted using TG analysis (TG, 209 F3 Tarsus, Selb, Germany). The pyrolysis was performed for all samples during three different heating rates: 5, 10, 20 K min^−1^, high purity nitrogen was used as the carrier gas with flow rate (15 °C/min), the samples weight was 3 mg.

**Figure 2:**
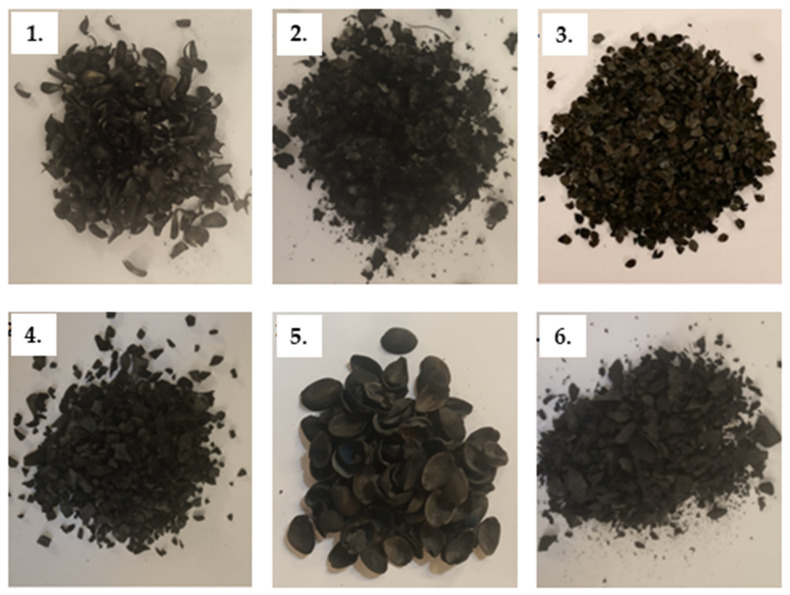
Pyrolysis product chars: sunflower hulls (**1**), corncobs (**2**), coconut (**3**), walnut (**4**), pistachio (**5**), buckwheat (**6**).

Once the pyrolysis was completed, the reactor with a sample was placed into an aluminium autoclave and cooled down to ambient temperature using neutral gas streamflow for immediate abort of pyrolysis to prevent the biomass from oxidising. The residuals were then divided into smaller samples and characterised (Figure 2). The CHNS-O analyser Flash 2000 (Thermo Scientific, Waltham, MA, USA) was used for conduction of elemental composition of the raw material and biochar. The non-organic fraction of samples was analyzed using X-ray fluorescence (XRF) analyzer Shimadzu 7000.

A solid fraction of pyrolysis process was subjected to chemical analysis. The heating values were measured in a calorimeter KL-12Mn. Proximate analysis involved the determination of the following parameters for the collected samples (using methods standardised by specified norms): moisture (CEN/TS 15414–1:2010; PN-EN 15414–3:2011), ash (PN-EN 15403:2011), volatile matter (PN-EN 15402:2011), and fixed carbon (by difference, with respect to other fractions). Volatile particles’ share in the material was measured after milling by placing 2 g samples in a melting pot under a cover of known mass and locating them in a furnace heated to 850 °C for 30 min. Once completed, the pot was weighed and the mass difference was registered. The same sample was heated at 815 °C for 2 h, but this time in the coverless pot for the sake of ash contribution measurement. The sample was then weighed once again. Three samples of each of the materials were examined according to the described procedure.

### 2.3. Proximal Analysis

The results from basic analysis of waste biomass properties, moisture, volatile matter, HHV, ash content and bounded carbon of raw materials with comparison to the example of other large biomass waste stream are presented in Table 2.

The buckwheat husks sample had the highest moisture content, while the pistachio husks sample had the least amount. Excluding pistachio husks, the values were similar for the other biomass samples, ranging from 8 to 10%. The highest volatile matter content of 85% had pistachio husks while the lowest of 74% had both coconut and buckwheat husks. Values differed noticeably and ranged from 74% to almost 86%. The ash content was almost the same for all specimens and was less than 1% of the mass percentage. The fixed carbon amount differed between biomass samples and ranged from 7% in sunflower husks to 16% in coconut husks.

The results of obtained by other authors are presented in Table 3. The aim of the research was to obtain high-quality solid fuel obtained from renewable sources. The basic parameter that should be compared with the previous research is the HHV of biomass, followed by its growth in biochars. Literature data confirm that the HHV of the tested biomass is similar. The results for different biomasses show how important the parameter of ash content is. Biomass with high mineral content, such as rice husk and corncobs, has a lower energy density. It should be noted that a large amount of ash that may be a problem in the use of boilers will be even more concentrated during pyrolysis, because the entire mineral part of the biomass remains in carbonization. The chlorine content in sunflower shells sample is 0.4 wt.%, and in the corn cobs is 0.3 wt.%.

The XRF analysis confirms that the presence of calcium (Ca), potassium (K), sulphur (S) and iron (Fe) in raw material have influence on the products composition in the combustion process (Table 4). Potassium and sodium evaporate during combustion and react with other exhaust gas components. As a result of this reaction, a low-melting product is formed, which settles on the walls of the apparatus. This is especially problematic in the case of the formation of deposits in the presence of chlorine compounds, as it significantly accelerates high-temperature corrosion. The composition of biomass ash is significantly different from that of typical fossil fuels (coal), especially with the highest contents of alkali metals. The analysed biomass contains a relatively small amount of sulphur compared to fossil fuels. It contributes to the formation of low-melting deposits in industry scale process, especially in the case of potassium forming eutectic deposits. Materials with a high alkali content are, among others, corn cobs, which is confirmed by the high potassium content (72.33% of the ash mass) after thermal degradation.

### 2.4. Theory of Kinetic Reaction of Pyrolysis Process

The pyrolysis process of biomass or other materials could be described using the various fitting model [46], which includes non-isothermal kinetic parameters for pyrolysis of solid materials and reaction order (*n*). The pyrolysis process is described as first order kinetic reaction (*n* = 1) and the basic Arrhenius Equation (1) was used to calculate reaction rate (*dx*/*dt*):

(1) $$\frac{dx}{dt}=k{\left(1-x\right)}^{n}$$

where: *t* is time (s), *x*—the conversion fraction of fuel sample (1), and *k*—rate constant (1/s) given by the Equation (2):

(2) $$k=Ae^{-\frac{E}{RT}}$$

where: *A* is pre-exponential factor, *E* is activation energy (J/mol), *T* is temperature (K) and *R* (J/(mol K) is the gas constant.

The definition of conversion fraction of the solid material/substrate in the pyrolysis process is the calculation as a function of mass sample, described by Equation (3):

(3) $$x=\frac{m_{0\;}-m}{m_{0\;}-m_{\infty }}$$

where *m*_0_ is the initial mass of the sample (g), *m*—the mass at time *t*, *m*_∞_—the mass at the final temperature.

During dynamic pyrolysis process data were obtained at a constant heating rate ($\beta =\frac{dT}{dt}$) and the first order reaction, which was inserted in Equation (1) and the transformation and rearranged expression into various methods described by Equations below (4)–(7).

- *Redfeld and Coast method* [47]

(4) $$\ln(-\ln\frac{\left(1-x\right)}{T^{2}})=\ln\frac{AR}{\beta E}\left(1-\frac{2RT}{E}\right)-\frac{E}{RT}$$

where A is the pre-exponential factor, and R the gas constant (J/molK). ln(β/T^2^_max_)

- *Kissinger method* [48]

(5) $$\ln(\frac{\beta }{T_{max}^{2}}\;)=\ln\frac{AR}{\beta E}-\frac{E}{RT_{max}}$$

where *T_max_* is the maximum of temperature peak (K)

- *Flynn-Wall-Ozawa (FWO) method* [49]

(6) $$\ln\beta =\ln\frac{AE}{R\;g\left(x\right)}-5.331-1.052\;\frac{E}{RT}$$

where *g*(*x*) is constant at a given value of conversion.

- *Kissinger-Akahira-Sunose (KAS) method* [50]

(7) $$\ln(\frac{\beta }{T^{2}}\;)=\ln\frac{AR}{Eg\left(x\right)}-\frac{E}{RT}$$

## 3. Results and Discussion

### 3.1. Thermogravimetric Analysis

**Figure 3:**
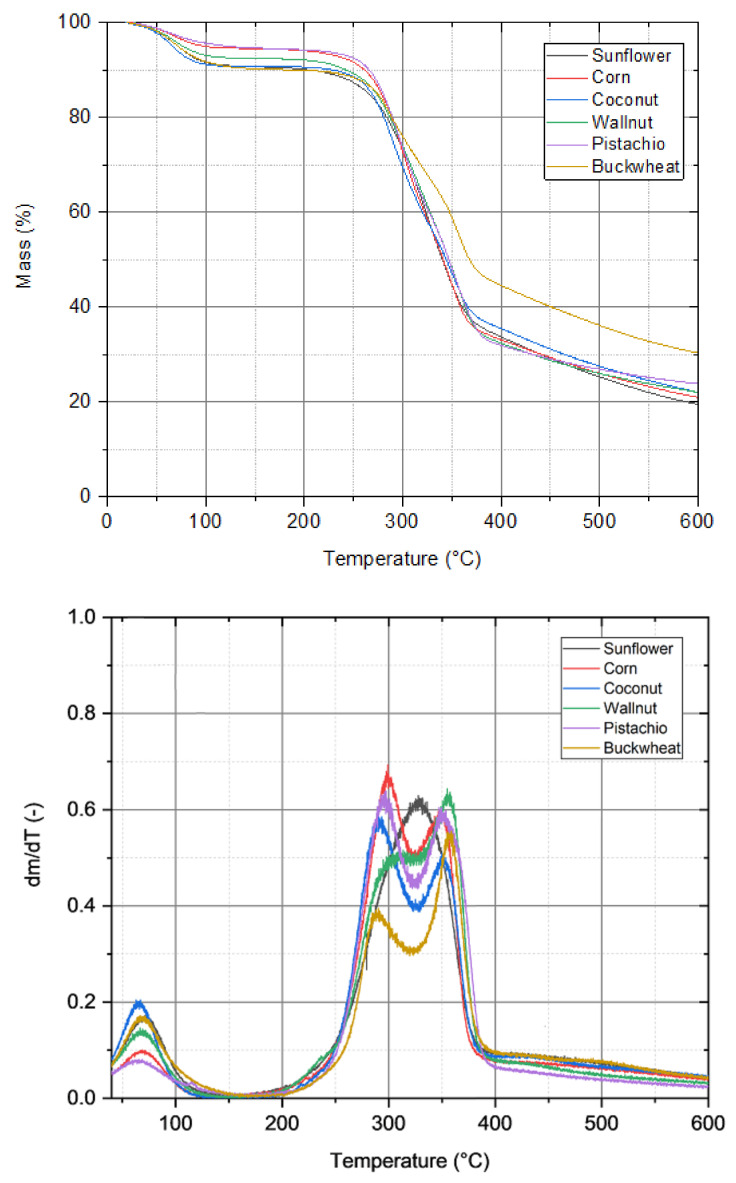
Thermogravimetric analysis of selected biomass samples.

Thermogravimetric analysis showed an overall similarity of the pyrolysis process for all specimens. Figure 3 shows a slight decrease of around 5–10% in the range from 0 to 100 °C, and an insignificant change of mass between 90 and 260 °C. The peaks below 100 °C for all samples are integrated with water evaporation. The most intensive degradation can be observed between 260 and 380 °C. The difference between the samples is the greatest in this range. Buckwheat husk lost approximately 45% of the mass, while the other samples lost almost 60%. A 20% loss of the mass was achieved in the following temperature interval between 380 and 790 or 850 °C. The analysis showed that buckwheat husks were the least susceptible to high temperatures and lost the least mass percentage, 73%, while sunflower husks lost the largest amount, almost 90%. The similar shape and intensity of peaks from degradation curves in TG analysis represent the same composition of raw materials. The left-hand peak is related to hemicellulose degradation, which is in the range of 250–350 °C [51]. The second peak in range of 320–400 °C is related to cellulose and lignin degradation, which causes the peak to extend. The intensity of peaks in buckwheat is smaller than in other biomass material, which could be caused by a smaller number of mentioned compounds. The sunflower degradation curve is different, which is associated with other components of raw material and could be caused by a high intensity of hemicellulose peak or cellulose without other components. The analysis of the degradation curves of raw materials confirm that the pyrolysis begins at about 250 °C and finished up to 400 °C, where the mass of samples is reduced to 30% of initial mass (the rest is the volatile matter). In this work, pyrolysis was conducted at 800 °C to completely remove volatile organic compounds to potential future use of char material as a calorific fuel-biochar means that the combustion process does not generate harmful exhaust gases, such as dioxins.

### 3.2. Kinetic Study

The kinetic analysis of pyrolysis degradation process was conducted for all biomass samples at three different heating rates, 5, 10, 20 K/min, and the results TG analysis were used in the calculation of energy activation for each biomass.

**Figure 4:**
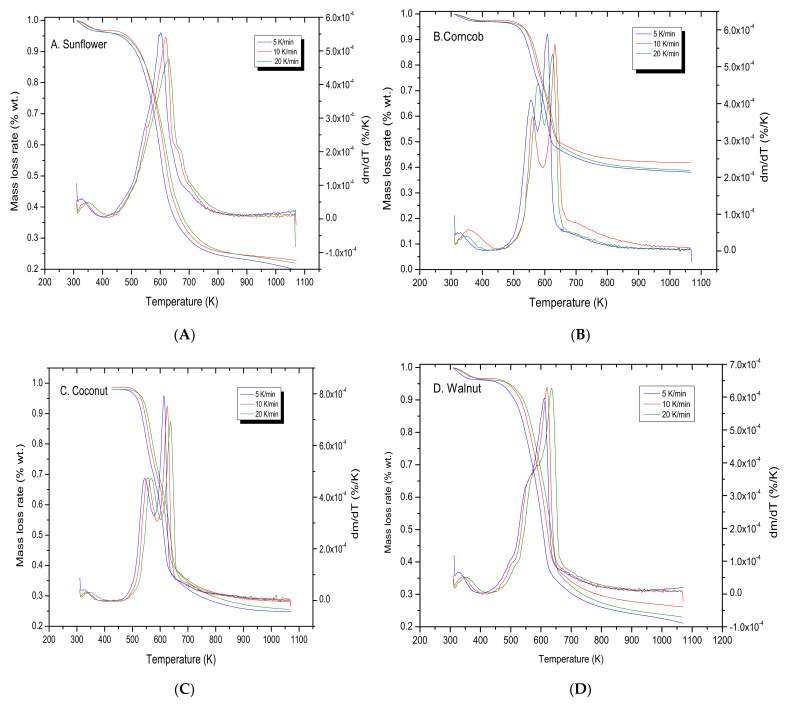

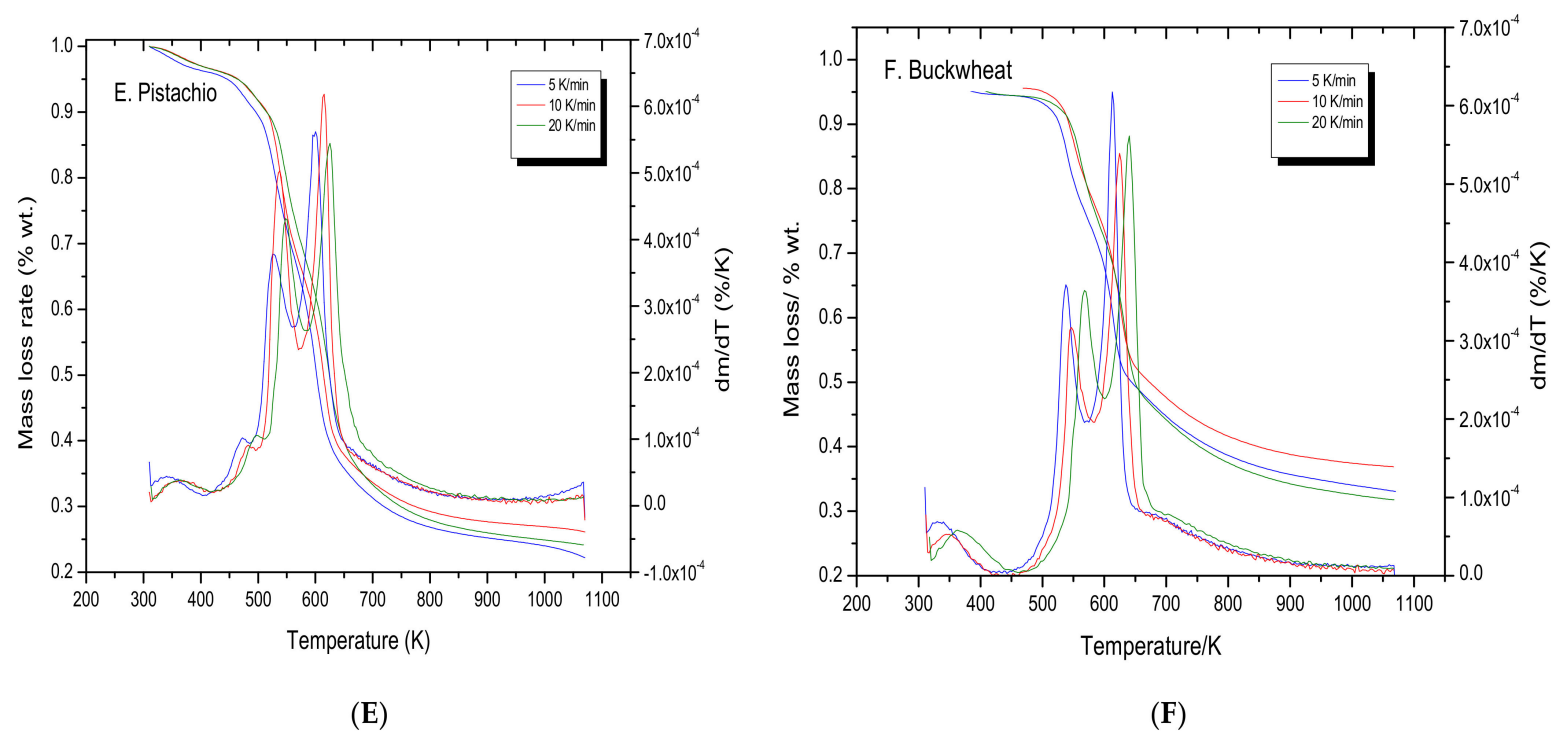
Biomass samples in TG analysis during various heating rates: 5, 10, 20 K/min. (**A**) Sunflower hulls, (**B**) Corncob, (**C**) Coconut shells, (**D**) Walnut husks, (**E**) Pistachio husks, (**F**) Buckwheat husks.

**Figure 5:**
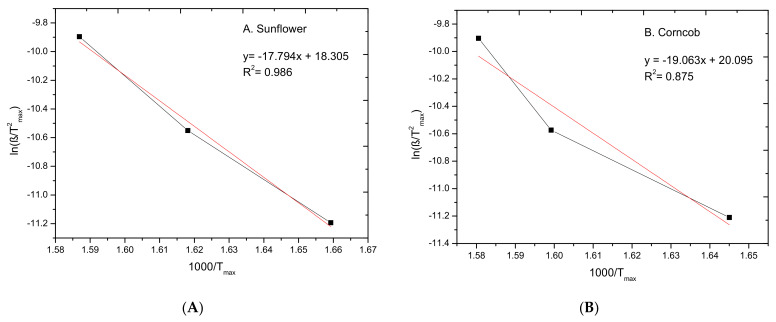

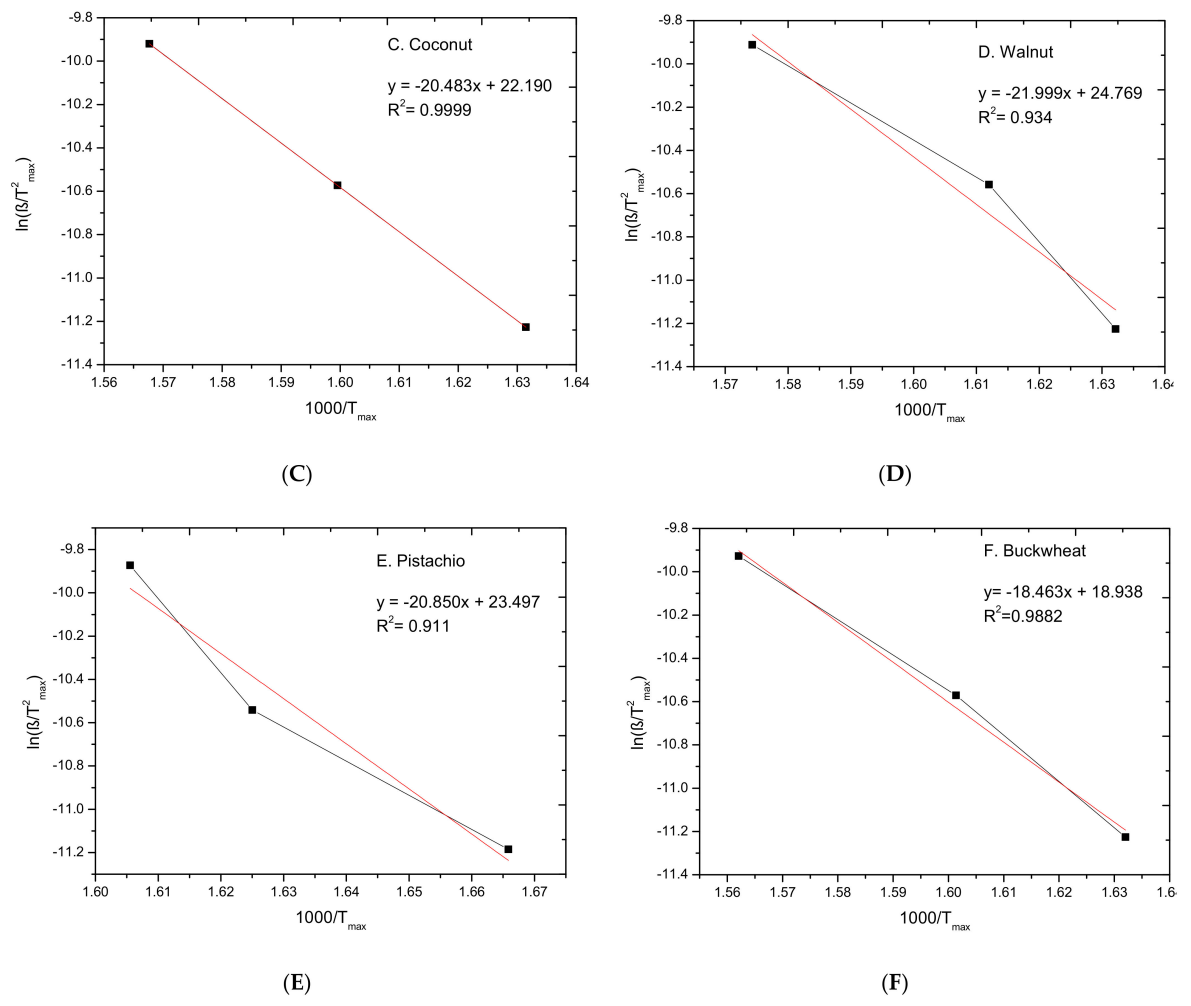
Kissinger plot of (**A**) Sunflower hulls, (**B**) Corncob, (**C**) Coconut shells, (**D**) Walnut husks, (**E**) Pistachio husks, (**F**) Buckwheat husks.

The Kissinger method for all biochar samples was used to calculate the energy activation of pyrolysis process in various heating rate. The energy activation was calculated from the plot of ln (β/T^2^_max_) against 1000/T_max_ for the series of experiments at different heating rates (β) for all biomass samples. The T_max_ is temperature which corresponds to the maximum of weight loss peaks—from the DTG curve for each heating rate (Figure 4). The data obtained during the degradation process were used in Equation (5) and are presented on Figure 5; the regression equations and the square of the correlation coefficient (R^2^) are presented on the plots.

The results obtained from the Kissinger method are presented in Table 5 for each sample, the activation energy (E_a_) and pre-exponential factor (A) calculated from the slope and intercept of plots. The activation energy is the minimum value of energy required to start a chemical reaction, therefore the more difficult the reaction to start the higher the activation energy value. An example of biomass with a high activation energy value is rice husk (206.8 kJ/mol) [52]. This material contains proportional contents of building polymers (31.2% cellulose, 22.5% hemicellulose and 22.3% lignin) [53,54,55].

The energy activation of sunflower sample (147.9 kJ/mol) is the lowest, and the DTG plots has only one characteristic peak. This could be interpretive as only one compound of biomass structure (hemicellulose, cellulose or lignin). The highest energy activation value (182.9 kJ/mol) was obtained for walnut sample. The range of activation energy values obtained was from 147.9 to 182 kJ/mol, which proves that the tested biomass, despite the differences in the structure and composition of compounds, is characterized by similar reaction kinetics and pyrolysis processes.

### 3.3. Properties and Elemental Analysis

The carbon content for all specimens was about 47% and hydrogen was around 6%. The nitrogen content differed noticeably: about 4% for pistachio, walnut, coconut and buckwheat and almost 1% for sunflower and corn. The calorific values of raw materials ranged between the minimum for corncob (15.8 MJ/kg) and maximum for walnut husks (18.7 MJ/kg). The calorific values obtained in the study were similar to the data presented by other authors, with the difference that according to the literature review, the material characterised by the lowest calorific value is buckwheat hulls (15.7 MJ/kg) [56], while the highest calorific value is characterised by walnut husks (18.9 MJ/kg) [41]. The results are similar in comparison with the reference value of 16.9 MJ/kg for commercial material (i.e., wood chips). The calorific values of the tested biomasses are higher than the widely analysed rice husk biomass of 13.24 MJ/kg [40], which represents a better energy quality of the tested materials. Moreover, rice husk is characterised by a much lower volatile matter content and a many times higher ash content. High ash content in biomass is undesirable and affects the lower energy value of the raw material. In addition, from a technological point of view, materials with a low ash content are sought after because it affects corrosion and pollution [57].

The HHV of examined biomasses and biochars was relatively similar, as shown in Table 6.

The minimum value of the combustion heat among the analysed materials was achieved for maize cobs (15.8 MJ/kg), while the maximum for walnut shells was (18.7 MJ/kg), which differs from the literature value by 2 MJ/kg [41]. This difference may be due to the different moisture content of the biomasses analysed, as the evaporation of the water contained in the material absorbs part of the energy generated in the combustion process, thus lowering the combustion heat value. Furthermore, the analysed biochars are characterised by higher combustion heat in comparison to the raw material samples. In addition, the biochar combustion heat values display greater standard deviation in comparison to those of biomasses. One of the advantages is the fact that char as a fuel is devoid of volatile organic compounds and is relatively environmentally friendly to burn. The average value of the heat of combustion increased due to pyrolysis process from 10 MJ/kg, with a minimal value of 2.7 MJ/kg for corncob and maximum of 13.0 MJ/kg for coconut, which is also characterised by the maximal absolute combustion heating value (32.3 MJ/kg). The increase in the calorific values varied from 15% to 172% (with 54% reference for wood chips), which indicates that charring is an effective method for increasing the energy density. Properties such as heat of combustion and content of the three most abundant chemical elements (i.e., carbon, nitrogen and hydrogen) of each biomass sample were compared with solid pyrolysis products.

The heat of combustion of biochar is on average 60% higher than for biomass. These values are similar to the calorific value of wood and wood charcoal, and some of them are fuels with higher calorific values (e.g., pistachio charcoal, walnut charcoal, and buckwheat charcoal). The advantage of the analysed biomass samples is their water content, which is several times lower than that of wood chips: higher contribution of water in the sample has a negative impact on the value of HHV. The low water content of the fuel during its harvesting is energetically beneficial (i.e., biomass does not require energy to be dried), it is also an advantage due to the lower susceptibility of dried biomass to microbial degradation.

**Figure 6:**
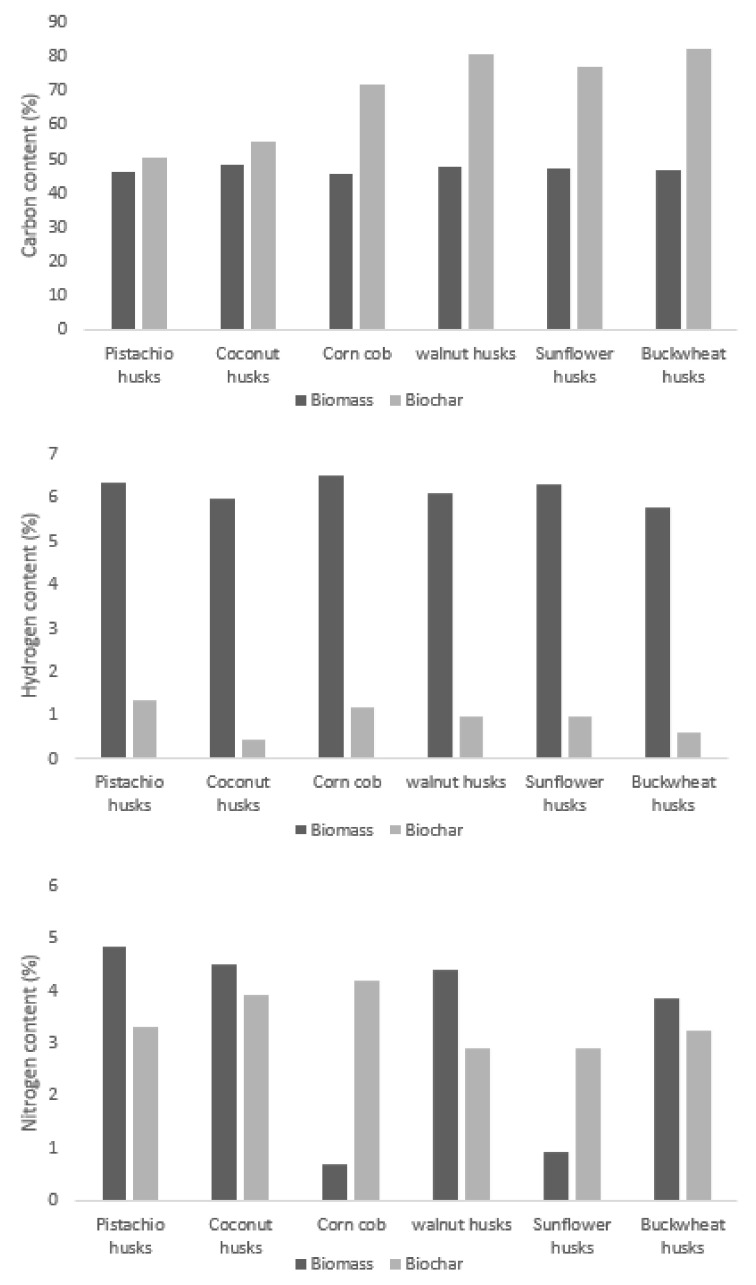
Carbon, Hydrogen and Nitrogen content in biomass and char from biomass.

The samples were subjected to elemental analysis. The carbon, hydrogen and nitrogen content in the samples subjected to pyrolysis and post-pyrolysis charcoal were determined (Figure 6). The carbon content for all types of biomass increased as a result of pyrolysis. The increase in carbon content varied from low for pistachios (4.2% wt.) and coconut (7% wt.) to the largest for buckwheat (35% wt.). The rise in carbon content increases the calorific value of the charcoal.

The share of hydrogen in pyrolysis products was several times lower than the share of this component in biomass. This effect is normal for biomass pyrolysis, which is a result of biomass degassing from cellulose to carbon chains. Low hydrogen content indicates degassing of the sample. Deprivation of volatile matter is beneficial for ecological combustion of carbonate in domestic boilers because it prevents tar generation.

The change in nitrogen content was not the same for all the pyrolyzed samples. The lowest nitrogen share in emissions are expected from walnut and sunflower husk combustion. Corncobs and sunflower husks present different behaviour in the process of pyrolysis: the change in nitrogen content is positive, which contrasts with the general trend in all other cases, where this value decreases.

Considering the sustainability point of view, the important characteristic of a fuel is the content of nitrogen in emissions, which is directly linked to nitrogen mass percentage. This observation can be explained by the probable lower rate of volatilisation of nitrogen-containing molecules during sunflower husk and corncob pyrolysis.

The main characteristic value for raw material samples is ash content, which is a representative value for comparing biomass-derived biochars. Ash contents per unit mass of biochar and unit heat of combustion were calculated for all the analyzed fuels.

**Figure 7:**
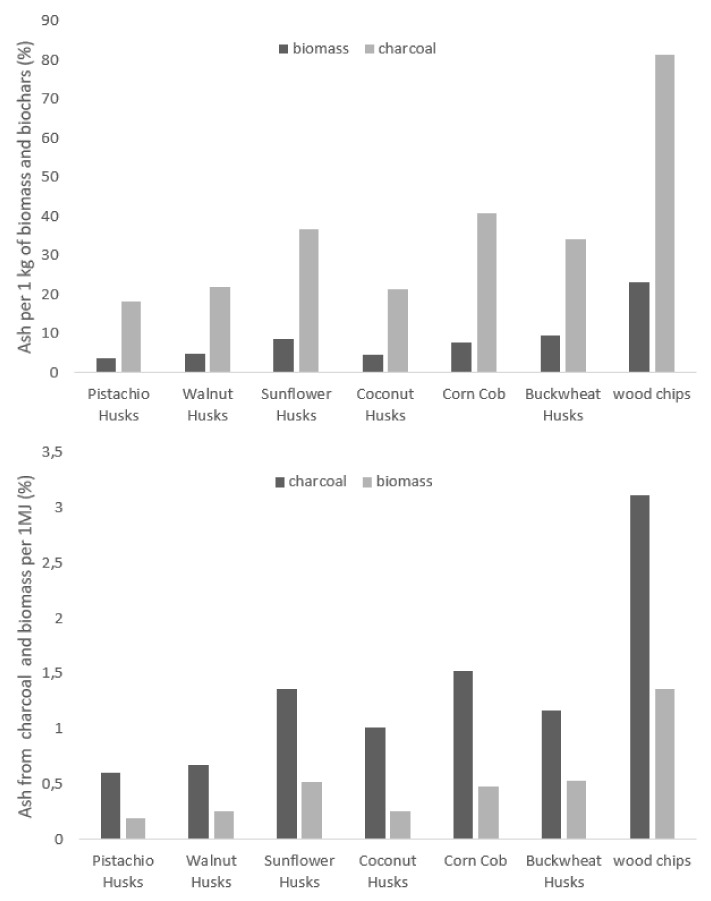
Amount of ash per 1 kg and one MJ of biomass and pyrolytic charcoal from biomass.

The summaries of the ash content per kilogram of biomass and kilogram of carbonate resulting from pyrolysis and per 1 MJ are presented in Figure 7. High ash content has negative impact for fuel quality [15]. Samples of waste biomass were combined with wood chips as an example of a popular biomass fuel. The results show that the types of biomass used in the research and the charcoal obtained from them are characterised by a low content of ash, which is a desirable property of fuel. The ash content for the given biomass ranged from 3.6 g for pistachios to 9.4 g per kg of biomass for buckwheat. It is several times lower ash content compared to woodchips, which contained 23 g of ash for 1 kg of fuel. A similar tendency was noticeable for charcoal, for which the ash content was like that of non-pyrolyzed wood chips and ranged from 18.2 g per 1 kg for pistachios to 40.1 g per 1 kg for corncob.

The data on ash content have been converted per unit of energy. This calculation aimed to facilitate easier comparison of fuels with different heat of combustion. It can be seen that the ash content per 1 MJ of energy ranges from 0.20 g for pistachios to 0.53 g per 1 MJ for buckwheat, with a much lower ash mass than for wood chips for which the ash mass per 1 MJ of energy is 1.36 g. After pyrolysis, the effect of low ash content is even more visible because carbonates from agro-waste are characterised by a high energy concentration. The ash content per 1 MJ for wood chips is 2.3 times higher for corncob and as much as 5.15 times compared to pistachio husks.

## 4. Conclusions

The results obtained in this study prove that the pyrolysis process could be an effective method for increasing the energy of biomass material (combustion heat) concentration in a unit of mass. The influence of the agriculture biomass type on the increasing energy concentration varied. Percentage-wise, augmentation of carbon share in total mass also differed between samples: the lowest mass contribution of this element among the materials studied in the paper characterised buckwheat and walnut husks, whereas pistachio shells displayed greatest mass share of carbon. Conversion of waste biomass into charcoal can be particularly beneficial when biomass production is highly concentrated in the areas where it is produced and will affect its export. A significant reduction in nitrogen per kilogram of fuel can be observed for most samples. The differences in carbon content between raw materials and char can be observed. The highest carbon content was in the buckwheat husk carbonate, which was 75% higher than the buckwheat husk. The lowest carbon content was in pistachio husk carbonate, which was only 8.9% higher than pistachio husk. The carbon content influence on quality of fuel and correlates with the calorific values of the samples. Charcoals obtained as a result of pyrolysis are characterised by much higher HHV and their calorific value is similar to hard coal and is much higher than wood’s calorific value.

The results were compared with wood chips, which are a widely used solid fuel of organic origin. The comparison confirms that the studied biomass-derived fuels are characterised by lower ash contribution than wood. An analogous observation was made for the obtained biochars: ash contribution was lower than for the chips, both for unit-mass and for unit-combustion-heat. The main advantage of this method is that it increases the calorific value and also bulk density of biomass char in comparison to raw material.

## Figures and Tables

**Table 1:** World production of selected biomass and its potential share in the energy sector.

Biomass	Production (Tg)	Waste from Crops (%)	Wastes (Tg)	Charcoal (Tg)	Energy from Waste (PJ)	Energy from Charcoal (PJ)	Char Equivalence of Carbon Mass (Tg)	Char Equivalence of Wood Mass (Tg)
Sunflower	52	30	15.6	2.55	246.48	68.99	2.37	3.51
Corncobs	850	13	110.5	20.6	1808.00	552	19.04	28.17
Coconut	60	35	21	5.38	371.70	158.11	5.45	8.07
Walnut	4	44	1.76	0.38	32.56	12.33	0.42	0.63
Pistachio	1.1	45	0.495	0.07	9.11	2.14	0.07	0.11
Buckwheat	2	38	0.76	0.19	14.21	4.08	0.14	0.21

**Table 2:** Composition and properties of the raw waste materials.

	Moisture Content (% wt.)	Volatile Matter (% wt.)	Ash Content (%mass)	Bound Carbon (%mass)	Calorific Value (MJ/kg)	Char Coal (%)
Sunflower husks	8.6 ± 0.14	83.6 ± 0.42	0.85 ± 0.00	6.9	16.4 ± 0.00	23.2 ± 0.00
Corncobs	9.0 ± 0.28	81.3 ± 0.14	0.76 ± 0.01	9.0	15.8 ± 0.14	18.6 ± 0.14
Coconut shells	9.0 ± 0.00	74.7 ± 0.00	0.46 ± 0.00	15.9	18.5 ± 0.07	21.5 ± 0.07
Walnut husks	8.4 ± 0.14	78.3 ± 0.28	0.48 ± 0.00	12.8	18.7 ± 0.00	22.1 ± 0.14
Pistachio husks	3.4 ± 0.00	85.6 ± 0.14	0.36 ± 0.01	10.7	18.4 ± 0.14	19.8 ± 0.14
Buckwheat husks	10.1 ± 0.14	74.4 ± 0.07	0.94 ± 0.01	14.6	17.7 ± 0.14	27.4 ± 0.14
Rice husks [40]	8.8	59.2	26.20	14.6	13.2	26.5

**Table 3:** The basic properties of biomass precursors from the literature.

Biomass	Heat of Combustion (MJ/kg)	Calorific Value (MJ/kg)	Ash Content (wt.%)	Volatiles (wt.%)	C	H	N	S	Cl	Ref.
Sunflower hulls	18.94	17.68	1.6	66.6	50.5	5.9	1.3	0.1	0.4	[41]
Corncobs	-	16.30	9.8	74.5	42.5	5.3	0.9	0.1	0.32	[41]
Coconut shells	-	17.35	0.6	79.2	47.9	6.4	0.1	-	-	[42]
Walnut husks	20.13	18.88	1.1	72.3	53.6	6.6	1.5	0.1	0.2	[41]
Pistachio husks	17.47	-	0.4	83.0	44.9	5.7	0.5	1.0	-	[43]
Buckwheat husks	17.19	15.72	2.7	84.2	41.8	5.2	2.5	0.11	0.01	[44]
Rice husks	13.78	-	5.9	65.1	38.6	5.0	1.4	0.03	-	[45]

**Table 4:** XRF analysis of biomass and biochars.

	K (wt.%)		Ca (wt.%)		S (wt.%)		Fe (wt.%)	
	Biomass	Biochar	Biomass	Biochar	Biomass	Biochar	Biomass	Biochar
Sunflower hulls	53.51	51.09	23.45	43.34	1.29	1.67	1.34	0.67
Corncobs	32.97	72.33	62.26	15.51	1.30	-	2.26	4.69
Coconut shells	37.95	72.29	25.31	22.23	1.40	-	4.08	4.61
Walnut husks	37.66	33.09	55.44	43.73	0.98	-	2.14	21.42
Pistachio husks	33.19	30.62	50.59	30.62	1.89	9.69	4.02	17.21
Buckwheat husks	52.37	-	33.47	-	2.10	-	3.08	-

**Table 5:** Energy activation (E_a_) and Pre-exponential factor (A) calculated using Kissinger methods.

Biomass	E_a_ (kJ/mol)	A (1/min)	R^2^
A. Sunflower hulls	147.9	0.089 × 10^9^	0.986
B. Corncob	158.5	0.533 × 10^9^	0.875
C. Coconut shells	170.3	4.33 × 10^9^	0.999
D. Walnut husks	182.9	57.0 × 10^9^	0.934
E. Pistachio husks	173.4	16.0 × 10^9^	0.911
F. Buckwheat husks	157.4	0.156 × 10^9^	0.988

**Table 6:** HHV of examined biomasses and respective biochars.

	HHV (MJ/kg)	
	Biomass	Biochar
Sunflower hulls	16.4	26.8
Corncob	15.8	27.0
Coconut shells	18.5	32.3
Walnut husks	18.7	21.2
Pistachio husks	18.4	30.1
Buckwheat husks	17.7	29.4
Rice husks [54,55]	13.8	-

## Data Availability

The data presented in this study is available on request from the corresponding author.
